# The role of iron uptake in pathogenicity and symbiosis in *Photorhabdus luminescens *TT01

**DOI:** 10.1186/1471-2180-10-177

**Published:** 2010-06-22

**Authors:** Robert J Watson, Peter Millichap, Susan A Joyce, Stuart Reynolds, David J Clarke

**Affiliations:** 1 Department of Biology and Biochemistry, University of Bath, Bath BA2 7AY, UK; 2 Department of Microbiology, University College Cork, Cork, Ireland

## Abstract

**Background:**

*Photorhabdus *are Gram negative bacteria that are pathogenic to insect larvae whilst also having a mutualistic interaction with nematodes from the family *Heterorhabditis*. Iron is an essential nutrient and bacteria have different mechanisms for obtaining both the ferrous (Fe^2+^) and ferric (Fe^3+^) forms of this metal from their environments. In this study we were interested in analyzing the role of Fe^3+ ^and Fe^2+ ^iron uptake systems in the ability of *Photorhabdus *to interact with its invertebrate hosts.

**Results:**

We constructed targeted deletion mutants of *exbD*, *feoABC *and *yfeABCD *in *P. luminescens *TT01. The *exbD *mutant was predicted to be crippled in its ability to obtain Fe^3+ ^and we show that this mutant does not grow well in iron-limited media. We also show that this mutant was avirulent to the insect but was unaffected in its symbiotic interaction with *Heterorhabditis*. Furthermore we show that a mutation in *feoABC *(encoding a predicted Fe^2+ ^permease) was unaffected in both virulence and symbiosis whilst the divalent cation transporter encoded by *yfeABCD *is required for virulence in the Tobacco Hornworm, *Manduca sexta *(Lepidoptera) but not in the Greater Wax Moth, *Galleria mellonella *(Lepidoptera). Moreover the Yfe transporter also appears to have a role during colonization of the IJ stage of the nematode.

**Conclusion:**

In this study we show that iron uptake (via the TonB complex and the Yfe transporter) is important for the virulence of *P. luminescens *to insect larvae. Moreover this study also reveals that the Yfe transporter appears to be involved in Mn^2+^-uptake during growth in the gut lumen of the IJ nematode. Therefore, the Yfe transporter in *P. luminescens *TT01 is important during colonization of both the insect and nematode and, moreover, the metal ion transported by this pathway is host-dependent.

## Background

*Photorhabdus *is a genus of Gram negative bioluminescent bacteria that are members of the Enterobacteriaceae and are therefore close relatives of important mammalian pathogens such as *Escherichia coli *and *Salmonella*. *Photorhabdus *have a complex life-style that involves a pathogenic interaction with insect larvae and a mutualistic interaction with nematodes from the family *Heterorhabditis (*for recent reviews see [1,2]). The bacteria can be normally found colonizing the gut of the infective juvenile (IJ) stage of the nematode. The IJ is a free-living, soil-dwelling stage of the nematode whose role is to seek out and infect susceptible insect larvae. Once inside the insect the IJ regurgitate their bacterial symbionts into the insect hemolymph and, here, the bacteria divide exponentially [3,4]. The bacteria produce a range of activities, including hydrolytic enzymes, that contribute to the efficient conversion of the insects internal organs and tissues into bacterial biomass and the insect eventually dies of septicemia 48-72 hours post-infection [5]. At this point the IJ recovers to become an adult hermaphrodite that feeds on the bacterial biomass and lays eggs that develop through juvenile stages (L1-L4) before adulthood. After 2-3 rounds of nematode reproduction uncharacterized environmental signals stimulate the formation of an alternative L3 stage nematode called the IJ. The IJ is initially colonized by 1-2 *Photorhabdus *cells in a complex transmission process that has only recently been phenomonologically described [6]. These founder cells grow and divide resulting in a final population of *Photorhabdus *in the IJ of between 50-100 colony forming units (CFU). The IJs then emerge from the insect cadaver ready to search for more susceptible insect larvae.

The *Heterorhabditis *nematode is bacteriophorous and, during growth and development, the nematode feeds on the bacterial biomass present within the cadaver. Therefore the *Photorhabdus *cells must be able to satisfy the nutritional requirments of the nematode population. The genetic basis of the nutritional interaction between *Photorhabdus *and *Heterorhabditis *is not well understood. There is some evidence that crystalline inclusion proteins (encoded by *cipA *and *cipB*) produced by *Photorhabdus *have a role in nematode nutrition. Mutations in the *cipA *and *cipB *genes of *Photorhabdus luminescens *NC1 resulted in a strain that was unable to support nematode growth and development [7]. However the *cipA *and *cipB *mutations were pleiotropic making it difficult to confidently define a role for these proteins in nematode nutrition. Nonetheless it has been shown that overproduction of CipA and CipB in *E. coli *can improve the growth and development of *Steinernema *nematodes implying some role for these proteins in nematode nutrition [8]. A mutation in another gene, *ngrA*, encoding a phosphopantetheinyl (P'pant) trabsferase, was also shown to prevent nematode growth and development [9]. The *ngrA *gene was shown to be required for the production of small bioactve molecules such as siderophores and antibiotics [9]. Interestingly the stilbene antibiotic produced by all strains of *Photorhabdus *(3,5-dihydroxy-4-isopropylstilbene (ST)) has been shown to be important as a signal for the nematode and is involved in stimulating the recovery of the IJ to the adult hermaphrodite [10]. Moreover we have also recently shown that a mutation in the *exbD *gene of *Photorhabdus temperata *K122 was unable to support the growth and development of its nematode partner, *H. downesi *[11]. The *exbD *gene encodes a component of the TonB complex which is important in mediating the active uptake of siderophore-iron complexes via their cognate outer membrane receptors [12,13]. The defect in symbiosis of the K122 *exbD *mutant was rescued by the addition of FeCl_3 _to the media suggesting that siderophore-mediated iron uptake was important for nematode growth and development [11].

Iron is an essential nutrient that is generally found in the insoluble ferric (Fe^3+^) form [14]. Many bacteria produce siderophores, molecules with very high affinities for Fe^3+^, in order to be able to successfully compete for Fe^3+ ^in their environments [15,16]. The siderophores bind the Fe^3+ ^and then bind to specific receptors on the surface of the bacteria. The siderophore-iron complex is then transported into the cell before the Fe^3+ ^is reduced to Fe^2+ ^and stored as a complex with iron-binding proteins such as bacterioferritin or used for the assembly of important cofactors such as Fe-S clusters [14,17]. Bacteria also have mechanisms to transport the low levels of ferrous (Fe^2+^) iron that may be available in their environments. These transport pathways include the FeoABC permease and the YfeABCD divalent cation transporter [14,18]. In this study we wanted to undertake a comprehenisive analysis of the role of iron in the symbiosis between the sequenced strain of *Photorhabdus *(*P. luminescens *TT01) and its invertebrate hosts i.e. the insect and the nematode partner, *H. bacteriophora*. Therefore we constructed targeted mutants in genes predicted to play important roles in the uptake of both Fe^3+ ^and Fe^2+ ^and we tested these mutants for their ability to interact with the different invertebrate partners of *Photorhabdus*.

## Results

### Genetic analysis of iron uptake systems in *P. luminescens *TT01

**Figure 1:**
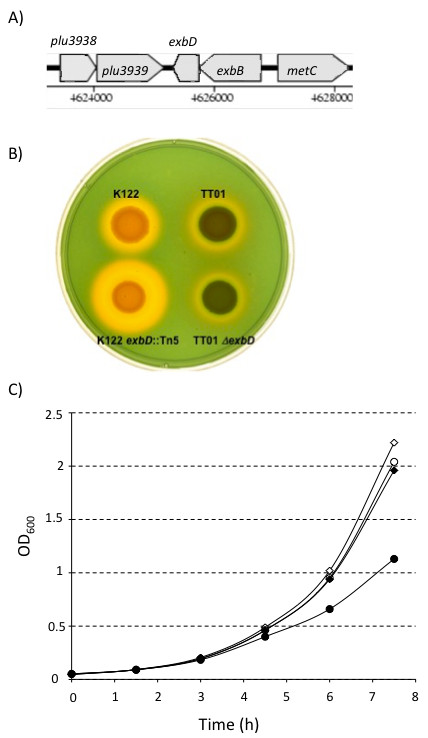
**The *exbD *mutant of *P. luminescens *TT01**. A) The *exbD *locus on the genome of *P. luminescens *TT01 (taken from Colibase at http://xbase.bham.ac.uk/colibase). B) Siderophore production by *P. temperata *K122, *P. temperata *K122 *exbD*::Km, *P. luminescens *TT01 and *P. luminescens *TT01 *ΔexbD*. The bacteria were cultured overnight at 30°C in LB broth and the OD600 of the culture was adjusted to 1. An aliquot of 10 μl of each cell suspension was inoculated onto the surface of an LB agar plate containing CAS solution and the plate was incubated at 30°C for 48 h. Siderophore production is observed as the orange halo surrounding the growing colony. C) The growth of *P. luminescens *TT01 *ΔexbD *is sensitive to the levels of iron in the medium. TT01 (diamonds) and the *ΔexbD *mutant (circles) were grown in fresh LB (open symbols) or LB broth supplemented with 50 μM 2'2'-dipyridyl (filled symbols). Growth curves were done in triplicate and a representative curve is shown.

We have previously shown that the *exbD *gene is important for both virulence and symbiosis in *P. temperata *(Pt) K122 [11]. The *exbD *gene encodes a component of the TonB complex (containing TonB, ExbD and ExbB) that is required for siderophore-mediated ferric (Fe^3+^) iron uptake in many bacteria [13]. The genome sequence of *P. luminescens *(Pl) TT01 has been available since 2003 at which time it was noted that the genome contained the largest known set of iron, heme, hemin and siderophore receptors [19]. This suggested an important role for iron acquisition in the life cycle of *P. luminescens *and we decided to undertake an analysis of the role of iron uptake in the sequenced strain. *In silico *analysis of the genome sequence of Pl TT01 identified a single *tonB *gene (*plu2485*) and a single genetic locus containing *exbD *(*plu3940*) and *exbB *(*plu3941*) (Figure 1A). To compare the role of the TonB complex in both Pl and Pt we constructed a deletion mutation in the *exbD *gene of Pl TT01 (the same gene that was mutated in Pt K122). It would be expected that the *ΔexbD *mutant strain would be crippled for iron uptake via any siderophore-mediated pathway. In Pt K122 the *exbD*::Km mutation resulted in an increase in the size of the halo produced on CAS indicator agar indicating accumulation of a siderophore in the agar ([11]and Figure 1B). We have previously shown that this siderophore is likely to be photobactin, a catechol siderophore that was originally identified in *P. luminescens *NC1 [11,20]. Although the Pl TT01 genome is predicted to encode a variety of siderophores, it is interesting that the *phb *genes, encoding the proteins required for photobactin biosynthesis, are not present [19]. Moreover, the Pl TT01 *ΔexbD *mutation was observed to have no affect on siderophore production as observed by no change in halo size on CAS agar (Figure 1B). Therefore, Pl TT01 does not appear to be limited for iron during growth on LB agar. Nonetheless we would expect that the *ΔexbD *mutant would be limited in its ability to scavenge for iron under iron-limiting conditions. To test this we cultured Pl TT01 and the *ΔexbD *mutant in LB supplemented with 50 μM 2'-2'-dipyridyl (DIP), an iron chelator, and measured growth (Figure 1C). In the absence of DIP, the growth curves of both the WT and the *ΔexbD *mutant were identical. However, in the presence of DIP, it was clear that the *ΔexbD *mutant grew at a slower rate than the WT confirming that the *ΔexbD *mutant was less efficient at scavenging iron.

**Figure 2:**
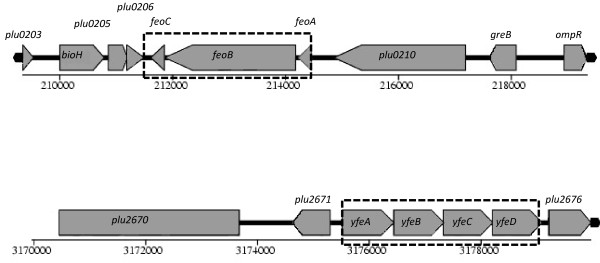
**The *feoABC *and *yfeABCD *loci in *P. luminescens *TT01**. The genetic loci predicted to encode the FeoABC permease and YfeABCD transporter (taken from Colibase at http://xbase.bham.ac.uk/colibase). The genes deleted in this study are highlighted with the dashed line boxes.

**Figure 3:**
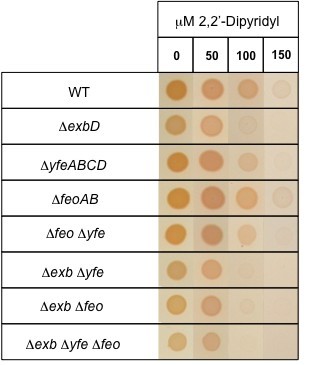
**The growth of *P. luminescens *in the presence of 2'2'-dipyridyl**. The sensitivity of each mutant to iron levels was assayed by determining the ability of each mutant to grow in the presence of increasing concentrations of 2'2'-dipyridyl. The OD_600 _of overnight cultures of each strain was adjusted to 1 and 10 μl of the cell suspension was spotted onto the surface of an LB agar plate supplemented with the indicated concentration of 2'2'-diyridyl. The plates were incubated at 30°C for 48 h before a digital photograph of each agar plate was taken. The final image was assembled by cutting and pasting the appropriate colony from each photograph using Adobe Photoshop 7. It is important to highlight that the photographs were not manipulated in any other way. The data shown is a representative example and the experiment was repeated in triplicate.

Bacteria can also utilize the small amounts of soluble ferrous (Fe^2+^) iron that are present in their environments, usually in a manner that is independent of the TonB complex. We identified genes encoding two potential TonB-independent Fe^2+ ^uptake systems, the FeoABC system and the YfeABCD system in the Pl TT01 genome (see Table 1 and Figure 2). The FeoABC system is encoded by the *feoABC *operon in which FeoB is predicted to be a GTPase directly involved in Fe^2+ ^transport [21]. On the other hand YfeABCD is an ABC transporter that mediates uptake of divalent cations, including Fe^2+ ^[18,22]. To test for the role of these genes in Pl TT01 we constructed *ΔfeoABC *and *ΔyfeABCD *mutant strains (*Δfeo *and *Δyfe *respectively). We also combined mutations to produce the double mutants *Δfeo Δyfe*, *ΔexbD Δyfe *and *ΔexbD Δfeo *and an *ΔexbD Δyfe Δfeo *triple mutant. These iron transport mutants were then tested for their ability to grow on iron-restricted medium i.e. LB agar supplemented with increasing levels of DIP. All strains could grow equally well in the absence of DIP and, as expected, all strains carrying the *ΔexbD *allele showed reduced growth, compared to the WT, on media containing 100 μM DIP (Figure 3). In addition, the *yfeABCD *locus may also play an important role in iron uptake as the *Δyfe *mutant did not grow as well as WT in the presence of 150 μM DIP. Moreover the affects of the *Δyfe *and *ΔexbD *mutations appear to be additive confirming that the Yfe ABC transporter and the TonB complex function independently (Figure 3). On the other hand, the *Δfeo *mutant was unaffected at all concentrations of DIP suggesting that this system does not play a significant role in iron scavenging under these conditions. Interestingly the *ΔexbD Δyfe Δfeo *triple mutant was still able to grow on LB agar plates (even in the presence of 50 μM DIP) suggesting that Pl TT01 has additional mechanisms for scavenging iron.

**Table 1:** Iron transport genes in *P. luminescens *TT01 analyzed in this study.

gene	Pl annotation	score	Best hit
*tonB*	*plu2485*	4e-27	PMI1355| tonB | P. mirabilis HI4320| TonB protein
*exbD*	*plu3940*	5e-68	YpsIP31758_0592| exbD | Y. pseudotuberculosis IP 31758
*exbB*	*plu3941*	1e-79	ECA0358| exbB | E. carotovora SCRI1043| Biopolymer transport
*feoA*	*plu0209*	8e-27	b3408| feoA | E. coli K12| Ferrous iron transport protein A
*feoB*	*plu0208*	0.0	b3409| feoB | E. coli K12| Ferrous iron transport protein B
*feoC*	*plu0207*	2e-20	ef| ZP_04612647.1| Yersinia rohdei ATCC 43380| FeoC
*yfeA*	*plu2672*	1e-136	YpsIP31758_1705| yfeA | Y. pseudotuberculosis IP 31758
*yfeB*	*plu2673*	1e-139	PMI1026| sitB | P. mirabilis HI4320| Iron ABC transporter
*yfeC*	*plu2674*	1e-124	YpsIP31758_1703| yfeC | Y. pseudotuberculosis IP 31758
*yfeD*	*plu2675*	1e-125	YpsIP31758_1702| yfeD | Y. pseudotuberculosis IP 31758

### Role of iron uptake in pathogenicity

**Figure 4:**
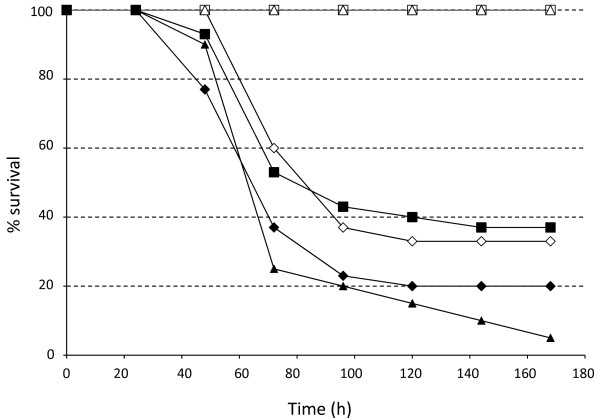
**Virulence of the *ΔexbD *and *ΔyfeABCD *mutants can be rescued by FeCl_3_**. Overnight cultures were prepared and 1000 CFU of WT (diamonds), *ΔexbD *(squares) and *ΔyfeABCD *(triangles) were injected into 5^th ^instar *M. sexta *larvae (open symbols) or larvae pre-injected with 10 μl of a solution containing 5 mM FeCl_3 _(filled symbols). The data shown is the mean of at least 2 independent experiments (with n = 10 insects/experiment). For clarity the standard deviations are not shown but these values were within expected limits (0-35%).

To determine the affect of the iron transport mutations on virulence we injected approximately 200 CFU of each strain into 10 *Galleria mellonella *larvae. Pl TT01 killed the insects in around 48 h, as did both the *Δyfe *and *Δfeo *mutant strains (data not shown). On the other hand no insects injected with the *ΔexbD *mutant died over the 168 h period of the experiment (data not shown). The *ΔexbD *mutant was also avirulent when injected into larvae of another insect model, the Tobacco Hornworm, *Manduca sexta *(Figure 4). Importantly, in *Manduca*, the virulence of the *ΔexbD *mutant could be rescued by the pre-injection of 5 mM FeCl_3 _into the insect (Figure 4). We have shown that the injection of 5 mM FeCl_3 _was not toxic to the insect (data not shown). Remarkably, whilst the *Δfeo *mutant was equally as virulent as the WT in *Manduca*, the *Δyfe *mutant was avirulent in this insect host (Figure 4). This suggests that the requirement of the *yfeABCD *operon as a virulence factor is dependent on the insect host. Moreover virulence of the *Δyfe *mutant could be rescued by the pre-injection of FeCl_3 _confirming that the ability to scavenge for iron is an important virulence factor in Pl TT01 (Figure 4).

### Role of iron uptake in symbiosis

**Figure 5:**
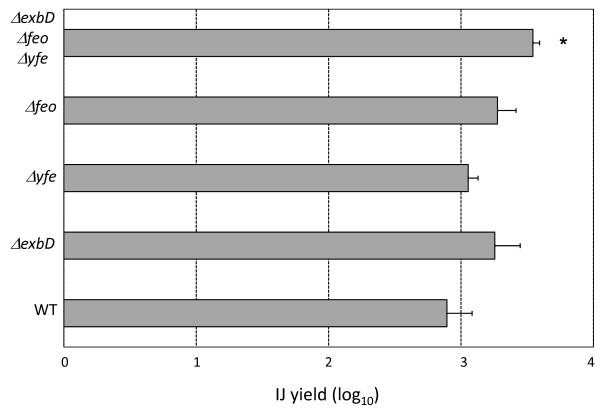
**The IJ yield after growth on *P. luminescens***. The different bacterial strains were inoculated onto lipid agar plates, incubated for 3-4 days at 30°C and 40 surface-sterilised *H. bacteriophora *IJs were added to the biomass. The plates were incubated for 21 days at 25°C and the IJ yields were determined (i.e. total number of IJs/50). For each experiment 5 plates were analyzed for each strain and the experiment was repeated 3 times. Therefore the data shown is the mean ± standard deviation of n = 15 plates for each strain. Statistical significance was determined using a T-test and IJ yields significantly different (P < 0.01) to those obtained using TT01 are indicated with an asterisk.

In this study we wanted to determine the affect of the iron homeostasis mutations in Pl TT01 on nematode growth and development. Therefore lipid agar plates were inoculated with the strains to be tested (Pl TT01, *ΔexbD*, *Δyfe*, *Δfeo*, *ΔexbD Δyfe Δfeo*) and, 4 days later, the bacterial biomass was seeded with 40 surface-sterilised *H. bacteriophora *IJs. We observed that all of the Pl TT01 mutants, even the *ΔexbD Δyfe Δfeo *triple mutant, were as competent as, if not better than, the WT in their ability to support the growth and development of their nematode partner as measured by the IJ yield i.e. total number of IJs collected/number of IJs inoculated (see Figure 5). This is in sharp contrast to what we had previously observed with Pt K122 *exbD*::Km where we reported that *H. downesi *nematodes failed to reproduce on the mutant bacteria [11].

**Figure 6:**
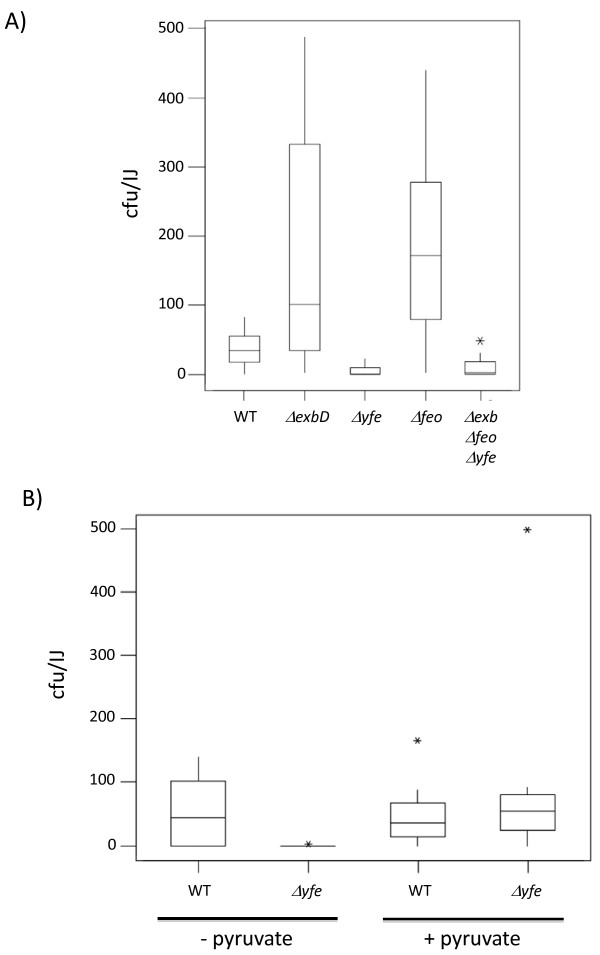
**Colonization of IJ nematodes with TT01 and mutant derivatives**. A) Individual IJ nematodes (n = 10), grown on the different bacterial strains (as indicated), were crushed and the lysate was plated on LB agar to enumerate the CFU within the nematode. The data is shown as a boxplot where the horizontal line within the box represents the median value. The box extends to the first and third quartiles and the whiskers show the upper and lower limits of the data (as defined by the statistical test). Asterisks represent outliers. The level of colonization of strains carrying the *ΔyfeABCD *allele was significantly lower than TT01 (P < 0.0001, Mann-Whitney). B) As above except that the lysate from each crushed IJ was plated on LB agar with or without added 0.1% (w/v) pyruvate, as indicated.

Nematode development culminates in the formation of a new generation of IJs that are colonized by the bacteria on which the nematodes have been cultured. Therefore, in order to ensure the symbiotic cycle had been completed, the IJs recovered from these symbiosis assays were surface sterilised, crushed individually and the lysate was spread onto LB agar. In this way it was determined that there were, on average, 42 CFU of Pl TT01 present in the gut of each IJ (Figure 6A). Moreover the *ΔexbD *and *Δfeo *mutant strains were able to colonize the IJ as well as the WT (Figure 6A). However the *Δyfe *and *ΔexbD Δyfe Δfeo *mutants appeared to colonize the nematodes at a level that was significantly lower than WT (P < 0.0001) suggesting that the *yfeABCD *locus may be important during colonization of the IJ (Figure 6A).

YfeABCD (also known as SitABCD) is an ABC divalent cation transporter that has been shown to transport both Fe^2+ ^and Mn^2+ ^[18,23,24]. In addition, both YfeABCD and Mn^2+ ^have been implicated in resistance to reactive oxygen species (ROS) [22,25]. *Photorhabdus *have been reported to be very sensitive to the low levels of ROS (particularly H_2_O_2_) generated in LB agar plates after exposure of the plates to fluorescent light [26]. Therefore the low numbers of CFU obtained with the *Δyfe *mutant could be explained by poor plating efficiencies due to an increased sensitivity to ROS. To test this we crushed IJs grown on either Pl TT01 or *Δyfe *and plated the lysate on LB agar supplemented with 0.1% (w/v) pyruvate (a known scavenger of H_2_O_2_). There was no difference in the number of WT Pl TT01 recovered from IJs when the lysate was plated on either LB agar or LB agar supplemented with pyruvate (Figure 6B). On the other hand, the number of CFU recovered from IJs grown on the *Δyfe *mutant increased to WT levels when the lysate was plated on LB agar supplemented with pyruvate (see Figure 6B). Similar results were obtained when the LB agar plates were supplemented with catalase (28 U ml^-1^) or if the plates were stored in the dark before use (data not shown). Therefore the *Δyfe *mutant does colonize the IJ to the same level as Pl TT01 although the *Δyfe *mutant appears to be more sensitive to ROS than the WT. Interestingly we did not see any difference in the sensitivity of WT or the *Δyfe *mutant to ROS when the strains were grown on LB agar and exposed to 30% (v/v) H_2_O_2 _(data not shown). Therefore the *Δyfe *mutant is not inherently more sensitive to oxidative stress and the increased sensitivity to ROS appears to be dependent on growth within the IJ, suggesting a role for the YfeABCD transporter in this environment.

### Bioassays using *H. downesi *reveals symbiosis defect in Pl TT01 *DexbD*

We had previously shown that the *exbD *gene in Pt K122 was required for the growth and development of *H. downesi *[11]. In this study we report that *H. bacteriophora *grows normally on the equivalent mutation in Pl TT01 (Figure 5). Therefore is the *H. downesi *nematode more sensitive to the *exbD *mutation or is the Pt K122 *exbD*::Km mutant less capable of supporting nematode growth and development in general? To test this we set up a set of bioassays whereby Pl TT01 *ΔexbD *and Pt K122 *exbD*::Km were incubated separately with their cognate nematode partner or the nematode partner of the other bacterium. For 14 days after inoculation we monitored nematode growth and reproduction and observed that *H. downesi *did not grow on the Pt K122 *exbD*::Km mutant, as expected, but did grow normally when cultured with Pt K122, Pl TT01 or Pl TT01 *ΔexbD *(Table 2). In contrast *H. bacteriophora *grew well on all strains tested suggesting that Pt K122 *exbD*::Km is not generally compromised in its ability to support nematode growth and reproduction. Therefore it does appear that the *H. downesi *nematode has a more stringent requirement for iron compared to *H. bacteriophora*.

**Table 2:** The growth and development of *Heterorhabditis *nematodes on cognate and non-cognate bacteria.

Bacteria	Nematode growth and reproduction^1^	
	
	*H. downesi*	*H. bacteriophora*
Pt K122	+	+
Pt K122 *exbD*::Km	-	+
Pl TT01	+	+
Pl TT01 *ΔexbD*	+	+

^1^presence (+) or absence (-) of nematode growth and reproduction after 14 days

## Discussion

In this study we have genetically tested the role of iron uptake in the interactions between *Photorhabdus *and its invertebrate hosts. We have constructed targeted deletions of genes on the *P. luminescens *TT01 genome that are predicted to be important in both ferric (Fe^3+^) and ferrous (Fe^2+^) iron uptake and we have tested these mutants for phenotypes associated with virulence against insect larvae and symbiosis with *H. bacteriophora *nematodes. Our results confirm that iron uptake is important during virulence of the insect and also reveal some interesting features of the role of divalent cation uptake during the pathogenic and mutualistic interactions of *Photorhabdus*.

In this study we have shown that the TT01 *ΔexbD *mutation is avirulent in the two different insect models that were tested. The *exbD *gene encodes for a protein that is part of the TonB complex that is found in many Gram negative bacteria. This inner membrane protein complex (composed of ExbD, ExbB and TonB) effectively transduces energy (using the proton motive force) from the inner membrane, across the periplasm, to the outer membrane [13,27]. The TonB complex interacts with outer membrane proteins (such as siderophore receptors) and the energy is used to facilitate the uptake of molecules through these outer membrane proteins. Bioinformatics can be used to identify proteins that interact with TonB based on the presence of a specific amino acid sequence called the TonB box. In this way 12 TonB-dependent receptors, the majority of which (75%) are predicted to be involved in iron uptake, have been identified in TT01 [27]. In this study we have shown that the lack of virulence associated with the *ΔexbD *mutation was due to the inability of this mutant to scavenge iron within the insect environment as virulence could be rescued by the pre-injection of FeCl_3_. Circulating iron in the insect is bound to transferrin and it has been shown that the transcription of the transferrin gene is increased in *M. sexta *after a microbial challenge suggesting that reducing the availability of iron is part of the insect innate immune response (P. Millichap, unpublished data). We have previously shown that a mutation in the *exbD *gene of Pt K122 was attenuated in virulence due to a decrease in the growth rate in the insect [11]. Our data suggests the Pl TT01 *ΔexbD *mutant strain is unable to grow in the insect implying that Pt K122 is better at scavenging iron in the insect. Although we have not investigated the reasons for this difference we have confirmed that, similar to what has been reported in other pathogens, TonB complex-mediated iron-uptake is critical for the virulence of *Photorhabdus*.

Nutritional interactions are one of the major driving forces in symbiotic associations [28-31] and our data suggests that iron is an important nutrient in *Photorhabdus-Heterorhabditis *interactions. During growth and development the nematodes feed on the bacterial biomass implying that this biomass must be able to satisfy all of the nematodes nutritional requirements, including the requirement for iron. We have previously shown that iron uptake in Pt K122 is required for the normal growth and development of Hd nematodes [11]. Therefore the Pt K122 *exbD::*Km mutant was not able to support Hd growth and development but this defect could be rescued by the addition of Fe^3+ ^to the media [11]. However, in contrast to this previous work, we have now shown that the *exbD *gene in Pl TT01 is not required for the normal growth and development of the Hb nematode. Cross-feeding experiments, where the Hb nematode was grown on Pt K122 and the Hd nematode was grown on Pl TT01, suggested that the nematode was responsible for this difference in iron dependency as the Hb nematode grew equally well on the Pt K122 *exbD*::Km mutant and the Pl TT01 *exbD *mutant. In addition, although the Hd nematode was observed to grow and develop on both Pl TT01 and the Pl TT01 *exbD *mutant, we did observe that the development of Hd IJ nematodes growing on the Pl TT01 *exbD *mutant was significantly delayed compared to Hb growing on the same bacteria (data not shown). This suggests that the Hd nematode might be more sensitive to the presence of the *exbD *mutation (and therefore iron levels) in their symbiotic bacteria. Such differences in sensitivity to iron levels may be one of the driving forces in the evolution and diversification of the *Photorhabdus-Heterorhabditis *system.

The FeoB protein is an inner membrane Fe^2+ ^permease that requires the FeoA-dependent hydrolysis of GTP [21]. The Feo transporter is present in many bacteria and has been reported to have a role in the anaerobic-microaerophilic environment of the gastrointestinal tract of mammals. In this study we show that the FeoABC transporter has no apparent role in either the pathogenic or mutualistic life-styles of *Photorhabdus*. The *yfeABCD *operon (also found in *Yersinia *and annotated as *sitABCD *in *Salmonella*, *Shigella *and avian pathogenic *Escherichia coli *(APEC) and *afeABCD *in *Actinobacillus*) encodes an ATP-dependent divalent cation transporter with affinity for Fe^2+ ^and Mn^2+ ^[32-36]. The Yfe/Sit/Afe transporter has been shown to have an important role in the virulence of many pathogens and a recent survey in *E. coli *has revealed a strong correlation between the presence of the *yfeABCD *operon and virulence [35]. In this study we have shown that the *yfeABCD *operon is important for the virulence of *P. luminescens *is some insect hosts. Therefore the *Δyfe *mutant was as virulent as the WT bacteria in one lepidopteran insect host, *G. mellonella*, but was completely avirulent in another lepidopteran host, *M. sexta*. This implicates the *yfeABCD *operon as a possible host-range determining locus in *P. luminescens*. The defect in virulence observed with the *Δyfe *mutant was rescued by the pre-loading the insect with Fe^3+ ^but not Mn^2+ ^suggesting that the role of the Yfe transporter in insect virulence is associated with iron homeostasis (data not shown).

In this study we have also shown that the Yfe transporter may have a role during the symbiotic interaction with the nematode, in particular during the colonization of the IJ. We observed that the *Δyfe *mutant has a very low plating efficiency, compared to WT, on LB agar when isolated directly from the IJ nematode. This low plating efficiency was rescued by the addition of either pyruvate or catalase, known scavengers of H_2_O_2_, to the LB agar plates. Therefore the *Δyfe *mutant appears more sensitive to H_2_O_2 _than the WT bacteria. The Yfe transporter can mediate the uptake of Mn^2+ ^and it has been shown that Mn^2+ ^can protect the cells from ROS [18,22]. Although it was thought that part of this protective affect was due to the ability of Mn^2+ ^to act as a chemical scavenger of ROS, recent evidence suggests that the role of Mn^2+ ^during oxidative stress in *E. coli *is as an enzyme co-factor (i.e. replacing the Fe^2+ ^in Fe-S clusters that are sensitive to oxidative stress) [25]. Many bacteria contain a dedicated Nramp-like Mn^2+ ^transporter called MntH [18,37]. In *E. coli *the expression of *mntH *can be induced by oxidative stress and it has been reported that *mntH yfe *double mutants in *Salmonella*, APEC and *Shigella *are sensitive to H_2_O_2 _[38-40]. Therefore Mn^2+ ^uptake appears to be critical in some cells for their ability to survive exposure to H_2_O_2_. Interestingly analysis of the Pl TT01 genome reveals that there is no *mntH *homologue in Pl TT01 and, therefore, the Yfe transporter is the only means by which Pl TT01 is predicted to be able to obtain Mn^2+ ^from the environment. However we could not detect any inherent increase in the sensitivity of the *Δyfe *mutant to H_2_O_2 _during growth on LB agar plates. This suggests that there is something specific about the conditions within the nematode that induces the H_2_O_2_-sensitive phenotype in Pl TT01 *Δyfe*. Recent studies in the model nematode *Caenorhabditis elegans *(a close relative of *Heterorhabditis*) have shown that this nematode produces 3 intestinally localized Nramp-like proteins that are involved in Mn^2+ ^transport from the gut lumen [41,42]. Therefore, the levels of Mn^2+ ^available to Pl TT01 within the gut of the IJ are likely to be very low. This, combined with the absence of a high-affinity Mn^2+ ^transporter, would be expected to result in a significant reduction in the level of intracellular Mn^2+ ^in the *Δyfe *mutant that would presumably also limit the ability of the bacterium to respond to an oxidative stress.

## Conclusion

In this study we show that siderophore-mediated iron uptake is important for the virulence of *P. luminescens *to insect larvae. This is similar to what has been reported for other pathogens and further highlights the relevance of *Photorhabdus *as a model for studying bacteria-host interactions [43]. Moreover, in contrast to what we previously reported in another species of *Photorhabdus *(*P. temperata *K122) [11], we show that siderophore-mediated iron uptake in *P. luminescens *TT01 is not required for the growth and development of the nematode. Therefore it appears that different *Photorhabdus-Heterorhabditis *complexes have specific requirements for iron. In addition we show that the *yfeABCD *operon (encoding the Yfe divalent cation transporter) is required for virulence in some, but not all, insect hosts. Although the Yfe transporter can mediate the uptake of either Fe^2+ ^or Mn^2+ ^we have shown that this transporter is involved in iron uptake during pathogenicity. On the other hand we present data that suggests that the Yfe transporter may be involved in Mn^2+^-uptake during growth in the gut lumen of the IJ nematode. Therefore, the substrate specificity of the Yfe transporter in *P. luminescens *TT01 appears to be dependent on the invertebrate host colonized by the bacteria.

## Methods

### Bacterial strains and growth conditions

Strains used in this study are listed in Table 3. *Photorhabdus temperata *K122, *Photorhabdus luminescens *subsp *laumondii *TT01 and *Escherichia coli *strains were routinely cultured in Luria-Bertani (LB) broth or on LB agar and were incubated at 30°C or 37°C respectively. CAS agar, for the detection of siderophores, was prepared by adding CAS solution (1:10 (v:v)) into the LB agar just before pouring. CAS solution was prepared as described previously [11]. When required antibiotics were added at the following final concentrations: kanamycin (Km) 50 μg/ml, ampicilin (Amp) 100 μg/ml, chloramphenicol (Cm) 20 μg/ml and rifampicin (Rif) 100 μg/ml.

**Table 3:** Bacterial strains used in this study

Strain	Genotype	Reference
*Photorhabdus*		
*P. temperata *(Pt) K122	Spontaneous Rif^R^mutant	Joyce and Clarke, 2003
*P. luminescens *(Pl) TT01	Spontaneous Rif^R^mutant	Bennett and Clarke, 2005
BMM417	K122 *exbD*::Km	Watson and Clarke, 2005
BMM430	TT01 *ΔexbD*	This study
BMM431 (*Δyfe*)	TT01 *ΔyfeABCD*	This study
BMM432 (*Δfeo*)	TT01 *ΔfeoABC*	This study
BMM433	TT01 *ΔexbD Δyfe*	This study
BMM434	TT01 *ΔexbD Δfeo*	This study
BMM435	TT01 *Δfeo Δyfe*	This study
BMM436	TT01 *ΔexbD Δfeo Δyfe*	This study
		
E.coli		
S17-1(*λpir*)	lysogenised with *λpir*, replication of *ori *R6K	Laboratory stock

### Construction of deletions in *exbD, feoABC *and *yfeABCD*

Targeted deletion mutants were constructed as previously described [10]. Briefly paired oligonucleotides are designed to amplify approximately 500-600 bp of DNA upstream (oligonucleotide 1 + 2) and downstream (oligonucleotide 3 + 4) of the gene to be deleted. The oligonucleotides used in this study are listed in Table 4. These amplicons, which have homologous terminal regions, are fused in a primerless PCR and ampliafied using oligonucleotide 1 +4 and then cloned into the suicide vector pDS132 [44]. After conjugation of the plasmid from *E. coli *S17-1 (λpir) into *P. luminescens *TT01 exconjugants were selected by growth in the presence of Cm and Rif. Potential mutants were then grown overnight in LB broth and plated on LB agar with 2% sucrose to select for loss of the plasmid via a second recombination event. Sucrose-resistant, chloramphenicol-sensitive colonies were then screened using colony PCR to identify mutants. Normally mutants are detected at a frequency of between 10-30% and the amplicons from 2-3 of the colonies are sequenced to confirm the integrity of the deletion.

**Table 4:** Oligonucleotides used for construction of targeted deletion mutants.

Gene(s)	Sequence 5' to 3'*	Name
*exbD*	**1**. TTATGCATGCGGTGATTGCTTCTGTTATTACTT GG	RJW115
	**2**. GAATCAGTGACAATTACATAAGTCACCTTGTCTTG	RJW116
	**3**. CAAGGTGACTTATGTAATTGTCACTGATTCTTCC	RJW117
	**4**. TTATGAGCTCGCCAACCAATTTGCCTCTGCCCTAC	RJW118
		
*yfeABCD*	**1**. TTATGCATGCGGTTATCAATACCTGCCAGATGC	RJW171
	**2**. CCCTTTTTGTTACATAAATTCAAACC	RJW172
	**3**. GGTTTGAATTTATGTAACAAAAAGGGTTATATCTG	RJW173
	**4**. TTATGAGCTCGGTGTTGAAGTTTGTTACTTATAGC	RJW174
		
*feoABC*	**1**. TTATGCATGCCGTAGTAAAAGCGGGTGATATCG	RJW167
	**2**. GCTAATCATTTTCAATTCCTACATATGACCTTCCG	RJW168
	**3**. CGGAAGGTCATATGTAGGAATTGAAAATGATTAGC	RJW169
	**4**. TTATGAGCTCCCAAAACGCTTCTCTTAGAAGATGC	RJW170

*: the underlined sequence indicate the restriction endonuclease sites used for cloning the amplicon into pDS132.

### Virulence assays

The pathogenicity of *P. luminescens *was assessed using final instar *Galleria mellonella *larvae (purchased from Livefood (UK)) and freshly molted 5^th ^instar *Manduca sexta *larvae (cultured at the University of Bath) as the model insect hosts. Briefly overnight cultures of *P. luminescens *TT01 were washed 3 times in 1 × PBS and the density adjusted appropriately so that 200 CFU or 1000 CFU could be injected into the hemolymph of *G. mellonella *or *M. sexta*, respectively. Insects were incubated at 30°C and monitored for death at regular time intervals. Where appropriate insect were pre-injected with 10 μl of either 5 mM FeCl_3 _or 5 mM MnCl_2 _at least 30 min before the bacteria were injected.

### Nematode growth and development

To determine the ability of each mutant to support nematode growth and development we carried out in vitro symbiosis assays. Therefore the bacteria were cultured overnight in LB and 50 μl was spread, in a Z pattern, onto the surface of a lipid agar plate (/500 ml: 12.5 g nutrient agar, 5 g corn syrup, 2,5 g yeast extract, 2.5 ml cod liver oil, 1 g MgCl_2_.6H_2_O) containing Rif and incubated at 30°C for 3-4 days. The bacterial biomass was then seeded with 40 surface-sterilised *H. bacteriophora *IJs and incubated at 25°C for 21 days. The presence of the Rif ensures that any bacteria present in the IJ are not able to compete with the lawn of bacteria present on the lipid agar plate. After 21 days the new generation of IJs had migrated to the lid of the Petri dish and these nematodes were collected in 1 × PBS and enumerated to determine the IJ yield (i.e. total number of IJs collected/number of IJs inoculated).

### Colonization assay

To determine colonization levels by each of the mutants IJs collected from the in vitro symbiosis assays were incubated at room temperature for at least 7 days before analysis. This incubation provides the bacteria with the opportunity to reproduce in the IJ gut and form a stable population. The IJs were surface-sterilised by washing in 0.4% (w/v) hyamine and individual IJs were crushed in 100 μl of PBS and the lysate was plated on LB (with or without added pyruvate). The plates were incubated at 30°C and the number of CFU's was determined after 48 h.

## Authors' contributions

RJW undertook all of the experiments described in this manuscript with the exception of the virulence assays in *Manduca sexta *(which were carried out by PM). RJW, SAJ and DJC conceived of the study. SAJ, SR and DJC designed the experiments and DJC wrote the manuscript. All authors have read and approved the final manuscript.
